# Impact of Wearing Face Masks on Patients with Severe Asthma During the COVID-19 Pandemic

**DOI:** 10.2147/JAA.S356912

**Published:** 2022-08-15

**Authors:** Kyoung-Hee Sohn, Myung-Nam Lee, Da Woon Sim, Sujeong Kim, You Sook Cho, Hyouk-Soo Kwon, Sang-Heon Kim

**Affiliations:** 1 Division of Pulmonology and Allergy, Department of Internal Medicine, Kyung Hee University Hospital, Seoul, South Korea; 2 Department of Nursing, College of Health Science, Kangwon National University Samcheok, Samcheok-si, Gangwon-do, South Korea; 3 Department of Allergy and Clinical Immunology, Chonnam National University Hospital, Chonnam National University Medical School, Gwangju, South Korea; 4 Division of Allergy and Clinical Immunology, Department of Internal Medicine, School of Medicine, Kyungpook National University, Kyungpook National University Hospital, Daegu, South Korea; 5 Department of Allergy and Clinical Immunology, University of Ulsan College of Medicine, Asan Medical Center, Seoul, South Korea; 6 Division of Pulmonary Medicine and Allergy, Department of Internal Medicine, Hanyang University College of Medicine, Seoul, South Korea

The novel coronavirus disease (COVID-19) affects millions of individuals worldwide, causing challenges in chronic non-communicable disease management. Previous reports show that 1.6–2.9% of COVID-19 patients have asthma and 2.8% of these patients led to poor outcomes of COVID-19.^1^ Vaccination for optimistic herd immunity, social distancing, and face mask usage are currently the main COVID-19 prevention strategies in many countries. South Korea has been affected by the pandemic since February 2020, and wearing face masks in public is mandatory. Although severe asthma patients are at a greater risk of severe COVID-19, no studies have yet assessed the effects of face masks on patients with severe asthma. Several countries have mandated or recommended wearing face masks despite the lack of evidence from randomized controlled trials. Furthermore, there is no action plan for wearing a mask in patients with chronic respiratory diseases and those with limited lung volumes, such as the elderly and pregnant women. Therefore, we assessed the 1) impact of the COVID-19 on severe asthma management and mask-wearing behaviors and 2) measured exercise capacity in severe asthmatic patients with and without a face mask.

Patients were recruited from five university hospitals in three different regions of Korea (Seoul, Daegu, and Gwangju) between August 2020 and December 2020 (Supplementary Figure 1). A total of 84 severe asthmatics were enrolled in this study (Supplementary methods).^2^ A questionnaire was developed by two quantitative research experts, and factors were evaluated on a 7-point Likert scale (0 = strongly disagree, 7 = strongly agree). The impact of wearing a Korean Filter (KF)-94 mask, which is equivalent to an American N-95 or an European filtering facepiece respirator 2, on exercise capacity were assessed by a 6-minute walk test (6MWT). Patients’ vital signs (saturation pulse oxygen [SpO_2_], heart rate, and Borg scale score for dyspnea) were measured without a KF-94 mask. After an hour of rest, the patients underwent another 6MWT while wearing a KF-94 mask and vital signs after 6MWT were re-measured. Supplementary Figure 2 shows a schematic illustration of the study design. Written informed consent was obtained from all participants before enrolment, and the study protocol was approved by the Institutional Review Board (Asan Medical Center IRB No.: 2020–1471). This study was performed in accordance with the Declaration of Helsinki.

**Figure 1:**
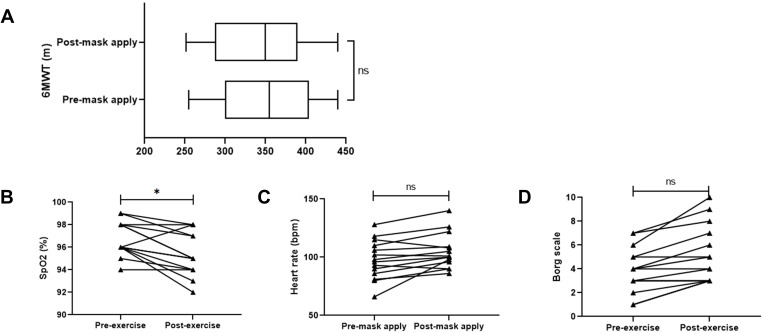
(**A**) Distance walked in the six-minute walking test (6MWT) by severe asthmatic patients with or without a KF-94 mask. (**B**–**D**) Comparison of changes in oxygen saturation (SpO_2_; (**B**), heart rate (**C**), and Borg scale score (**D**) before and after 6MWT between the two conditions. *P<0.05, ns: not statistically significant. GraphPad Prism 7 (GraphPad Software, CA, USA) was used for statistical analyses.

We surveyed a 7-point Likert scale questionnaire to 84 participants (mean age: 57.7 ± 11.4 years). Supplementary Table 1 shows the patients’ sociodemographic and clinical data. The Likert scale score for asthma exacerbation with mask use was 3.98±1.04, indicating a neutral response (Table 1). Notably, 71.4% (60/84) of patients responded that asthma was better controlled in this pandemic year of 2020 than in the previous year, and 23.8% (20/84) experienced asthma exacerbation during the pandemic year. Additionally, 22.6% of the patients were re-scheduled to later outpatient clinic appointments throughout the COVID-19 pandemic. Almost twice as many patients experienced delayed outpatient clinics in areas with a high incidence of COVID-19 (such as Daegu, a special pandemic control area) compared to the less afflicted areas. The most common reasons included “worried about cross-infection of COVID-19 in the hospital” (84.2%, 16/19), “effort to socially distance” (10.5%, 2/19), and “asthma was controlled well” (5.3%, 1/19). The 6MWT distance without a mask was 353 ± 58 m, which was not significantly different from that while doing the 6MWT after wearing the mask (342 ± 57 m, *P* = 0.659, Figure 1). Patients exhibited marginal yet significantly lower SpO_2_ with masks than without masks (95.3% vs 96.8%, *P* = 0.043), but heart rate and Borg scale ratings were not significantly different between the two conditions.

**Table 1:** Survey on Mask Use Behavior and Perception of Patients during the COVID-19 Pandemic

Question	Response (n, %)
Q1: Have you experienced an exacerbation of asthma during COVID-19 outbreak?* (yes, %)	20 (23.8%)
**Q2: Do you feel that your asthma is better controlled (on average) during the COVID-19 pandemic compared to that in the previous years?**	60 (71.4%)
**Q3: Have you experienced an exacerbation of asthma from wearing a mask during outdoor activities due to COVID-19?**	2.24 ± 2.07
Lung function FEV1/FVC < 70% (n=35): 2.69 ± 2.05	
Lung function FEV1/FVC ≥ 70% (n=48): 2.02 ± 2.04	
**Q4: Are you worried that you may be develop COVID-19 during the pandemic?**	2.73 ± 2.14
COVID-19 pandemic epidemic region (n=10): 3.20 ± 1.89 COVID-19 pandemic non-epidemic region (n=20): 2.35 ± 2.31	
**Q5: Have you delayed scheduled outpatient clinic appointments during the COVID-19 pandemic?**	19 (22.6%)
COVID-19 pandemic epidemic region (n=10): 3 (30%)	
COVID-19 pandemic non-epidemic region (n=20): 3 (15%)	
**Q5-1^†^: What was the main reason for delaying your medical appointments during the COVID-19 pandemic?**	
1) Worried about cross-infection of COVID-19 in the hospital: 16 (84.2%)	
2) Effort to socially distance: (10.5%)	
3) Asthma was controlled well: 1 (5.3%)	

**Notes**: Data are presented as numbers (percentages) or mean ± SD. (Likert 7-point scale: 1=strongly disagree, 7=strongly agree). COVID-19, coronavirus disease 2019; FEV1/FVC, forced expiratory volume in 1 s/forced vital capacity. *February 2020 to December 2020. ^†^Patients who answered yes to question 5.

A previous study reported that N-95 masks cause hypoxic or hypercapnic respiratory failure in chronic obstructive pulmonary disease patients with forced expiratory volume in 1s < 30%.^3^ In our study, the most commonly used masks by patients with severe asthma were the KF-94, surgical, and cloth masks, in the mentioned order (Supplementary Table 2). The World Health Organization recommends an N-95 mask for healthcare workers only during the COVID-19 pandemic.^4^ A recent study has shown that surgical masks can sufficiently protect against COVID-19 RNA aerosol transmission.^5^ Although wearing the KF-94 mask statistically lowered the saturation after 6MWT, it was still within the normal physiologic range. Severe asthmatic patients should be informed that surgical masks are available if they experience dyspnea and headaches with an N-95 mask.

Interestingly, more than two-thirds of the patients reported that their asthma was well controlled during this pandemic year of 2020 than in the last year. During the COVID-19 pandemic year, the exacerbation rate was lower than that of a similar out multicenter cohort in previous years (23.8% vs 41.0%).^6^ Our results are consistent with recent literature shows that the COVID-19 pandemic did not affect asthma exacerbation in general asthma patients.^7^ Furthermore, recent study reported that COVID-19 pneumonia did not induce severe asthma exacerbation.^8^ Although several hypotheses have been raised, this may be attributed to reduced exposure to outdoor allergens and decreased viral infection secondary to improved personal hygiene from face mask usage.

Our study had some limitations. This descriptive study focused only on patients with severe asthma, without the comparison of a control group. Moreover, the effects of KF-94 masks on lung physiology were assessed using the 6MWT, limiting the generalizability to real-world activities.

Since the outbreak of the COVID-19 pandemic, severe asthma patients were likely anxious for COVID-19 infection, but their symptoms of asthma were relatively well-controlled in real-world. Patients with severe asthma demonstrated that exercise capacity and subjective breathlessness did not increase significantly during the 6 MWT, although oxygen saturation was slightly lower with N-95 masks than without the masks. Further studies on the individual strategies for adherence to face mask usage and their efficacy in patients with severe asthma are necessary.
